# Division of Labor Associated with Brood Rearing in the Honey Bee: How Does It Translate to Colony Fitness?

**DOI:** 10.1371/journal.pone.0016785

**Published:** 2011-02-08

**Authors:** Ramesh R. Sagili, Tanya Pankiw, Bradley N. Metz

**Affiliations:** 1 Department of Horticulture, Oregon State University, Corvallis, Oregon, United States of America; 2 Department of Entomology, Texas A&M University, College Station, Texas, United States of America; Cajal Institute, Consejo Superior de Investigaciones Científicas, Spain

## Abstract

Division of labor is a striking feature observed in honey bees and many other social insects. Division of labor has been claimed to benefit fitness. In honey bees, the adult work force may be viewed as divided between non-foraging hive bees that rear brood and maintain the nest, and foragers that collect food outside the nest. Honey bee brood pheromone is a larval pheromone that serves as an excellent empirical tool to manipulate foraging behaviors and thus division of labor in the honey bee. Here we use two different doses of brood pheromone to alter the foraging stimulus environment, thus changing demographics of colony division of labor, to demonstrate how division of labor associated with brood rearing affects colony growth rate. We examine the effects of these different doses of brood pheromone on individual foraging ontogeny and specialization, colony level foraging behavior, and individual glandular protein synthesis. Low brood pheromone treatment colonies exhibited significantly higher foraging population, decreased age of first foraging and greater foraging effort, resulting in greater colony growth compared to other treatments. This study demonstrates how division of labor associated with brood rearing affects honey bee colony growth rate, a token of fitness.

## Introduction

Division of labor is one of the key features that have contributed to the great ecological success of social insects [1], [2]. Honey bee workers perform different tasks as they age and this phenomenon is referred to as temporal polyethism or division of labor [3], [4], [5]. After emergence as adults, usually the worker bees first clean cells, and as they age they feed the larvae and queen, process and store food, secrete wax and construct comb, and guard the entrance. The most prominent behavioral change is observed when the bees are about three weeks old, the age when they start foraging [6], [7]. Plasticity is an important attribute of division of labor and colonies respond to changes in the internal and external environment by manipulating the ratios of individual workers involved in different tasks [3]. Such plasticity in division of labor can be partially attributed to behavioral flexibility of the individual workers [3].

Division of labor is also observed during brood rearing in honeybees. Brood rearing in honey bees is accomplished by the combined labor of nurse and forager bees that directly or indirectly provision larvae, respectively. Pollen and nectar are the two primary resources for which bees forage. Nectar serves as a carbohydrate source for both adults and larvae, whereas pollen is the primary source of protein. Pollen is consumed by nurse bees that biosynthesize proteinaceous glandular secretions that are progressively provisioned to larvae [8]. Through the nurses, larvae are the major consumers of pollen in the colony. Honey bee colonies respond to amount of larvae present in the colony by adjusting the number of pollen foragers and individual pollen forager effort. More larvae result in a greater proportion of pollen foragers [9].

Pheromones play a significant role in honeybee division of labor. Queen mandibular pheromone (QMP) and brood pheromone (BP) have been shown to influence division of labor in worker honey bees [10], [11], [12]. Brood pheromone (BP) is a 10-component mixture of fatty acid esters extractable from the surface of honey bee larvae [13]. Brood pheromone communicates presence of larvae and their numbers to adult bees in a colony. Brood pheromone treated colonies rear significantly greater amounts of brood, have significantly higher ratios of pollen to non-pollen foragers, foragers return with heavier pollen loads and take more foraging trips per unit time, and age of first foraging is significantly lower [14], [9], [15], [16], [17], [18].

Studies into the effects of brood pheromone have generally avoided examination of the interaction between applied dose and pheromone effect. Thus studies related to effect of dose are extremely important to our understanding of pheromonal regulation of colony level foraging behavior. Results from LeConte et al. (2001), show that brood pheromone effect on foraging ontogeny is related to dose such that a relatively high dose increases age of first foraging, while a relatively low dose decreases age of first foraging. Thus, brood pheromone acts in a dose-dependent manner to alter the demographics of colony foraging behavior. Brood pheromone influences suites of foraging and brood rearing behaviors and as such may serve as a powerful tool to alter the foraging stimulus environment and thus change honeybee foraging strategies [10], [12], [19], [14], [16]. Addition of a relatively low dose of brood pheromone results in increased brood rearing and colony growth, factors that are directly related to reproduction and therefore fitness [14]. In this study we used different doses of brood pheromone to alter the foraging stimulus environment, thus changing the demographics of colony division of labor. We examined the effects of two different doses on individual foraging ontogeny and specialization, on colony level foraging behavior, and on individual protein synthesis, all critical aspects of within-nest care and outside provisioning of brood.

Colony growth and reproduction are principal sources of fitness for individuals in a social insect colony. In honeybees, colony growth is achieved through increased brood rearing. Also, genetic diversity promotes colony growth in honey bees [20]. In sharp contrast to the number of empirical studies on division of labor and individual foraging effort, there is a paucity of studies demonstrating how various foraging strategies affect an important life history trait, such as brood rearing. To place colony foraging strategies in both an evolutionary and apicultural context, there is need to understand how different strategies affect colony growth. Brood pheromone is an excellent empirical tool for altering various honey bee foraging strategies.

Division of labor is widely proclaimed as benefitting fitness. Changes in individual and colony behaviors in response to changes in colony state have been studied extensively and various models have been used to investigate mechanisms involved in efficient task allocation, but how these behavioral changes ultimately affect colony fitness have not received much attention. Here we attempt to investigate and demonstrate how division of labor associated with brood rearing affects honey bee colony growth rate, a fitness trait, by manipulating brood-rearing division of labor using brood pheromone.

## Results

**Figure 1:**
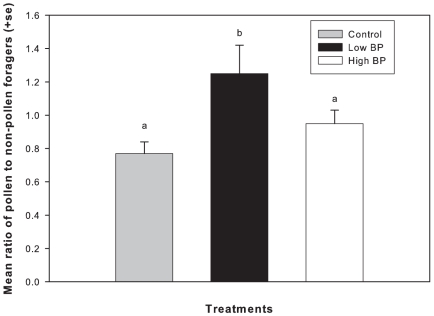
Mean ratio of pollen to non-pollen foragers (+SE). Different letters indicate significant differences between treatments. 3×2 contingency table analysis was used (Chi-square, P<0.001).

**Figure 2:**
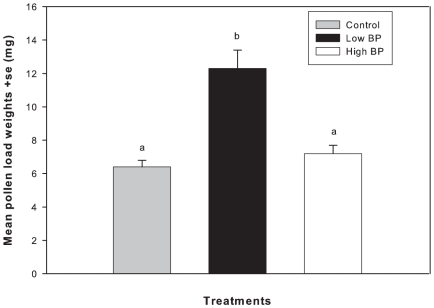
Mean pollen load weights collected by the foragers (+SE). Different letters indicate significant differences between treatments. ANOVA was used to analyze data, P<0.001. N = 400 bees per treatment. Tukey's HSD was used for pairwise comparisons (P<0.05).

The ratio of pollen to non-pollen foragers entering the colonies in an interval of 5 minutes was significantly greater in Low BP treated colonies throughout the experimental period (3×2 contingency table analysis χ^2^ = 81.5, 2df, P<0.001) (Fig. 1). Ratio of returning pollen to non-pollen foragers did not significantly differ among control and High BP treated colonies (3×2 contingency table analysis, P>0.05). Bees in Low BP treated colonies returned with significantly heavier pollen loads than control and High BP treated colonies (F_2,12_ = 14.3, P<0.001) (Fig. 2), and there was no significant difference in the pollen loads returned among High BP treatment colonies and controls.

**Figure 3:**
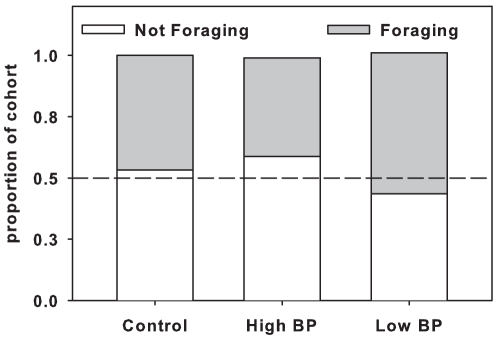
Mean proportion of foragers and non-foragers in each of the three treatments. 3×2 contingency table analysis was used (Chi-square, P<0.01).

**Figure 4:**
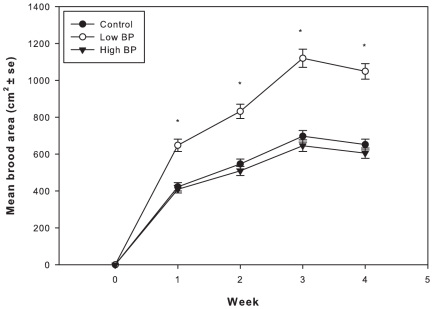
Mean brood area in cm^2^ (+SE). Asterisks indicate significant difference. Repeated measures ANOVA was used to analyze the data, P<0.001. N = 40 observations per treatment. Tukey's Post-hoc test used (P<0.05).

The proportion of foragers and non-foragers were significantly different among the treatments (3×2 contingency table analysis χ^2^ = 29.3, 2df, P<0.01). Low BP treatments had higher proportion of foragers followed by control and High BP treatments (Fig. 3). Low BP treated colonies reared significantly more brood area than High BP treatment colonies and controls (repeated measures F_2, 12_ = 19, P<0.001) (Fig. 4). There was no significant difference between the brood areas reared by High BP and control colonies (P>0.05).

**Figure 5:**
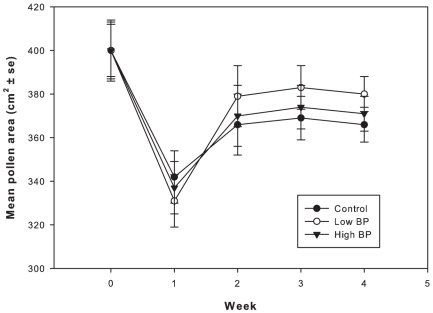
Mean pollen area in cm^2^ (+SE) for the three treatments. Repeated measures ANOVA was used to analyze the data, P = 0.3. N = 40 observations per treatment.

**Figure 6:**
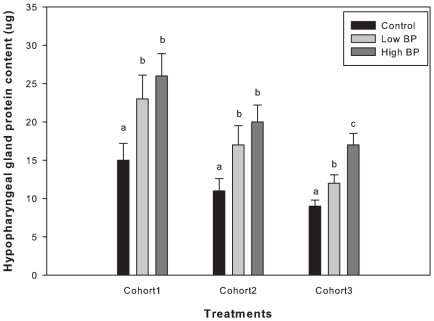
Mean hypopharyngeal gland protein content in micro grams (+SE). Different letters indicate significant difference. ANOVA was used to analyze the protein data, P<0.001. N = 200 bees per treatment. Tukey's HSD used for pairwise comparisons (cohort 3: P<0.05).

Amount of stored pollen was not significantly different among treatments during all the four weeks (repeated measures F_2,12_ = 1.3, P = 0.3) (Fig. 5). Hypopharyngeal gland protein content of bees analyzed from cohort 1 was significantly lower in the control treatments compared to High BP and Low BP treatments (Fig. 6), and there was no significant difference between the High and Low BP treatments (P>0.05). Similar results were obtained for bees obtained from cohort 2 with respect to hypopharyngeal gland protein content. Hypopharyngeal gland protein content of bees from Cohort 3 was significantly different among the three treatments with Low BP treatments having highest protein content followed by High BP and controls respectively (P<0.001) (cohort 1: df = 2, F = 26.7, P<0.001; cohort 2: df = 2, F = 39.5, P<0.001; cohort 3: df = 2, F = 24.8, P<0.001).

**Figure 7:**
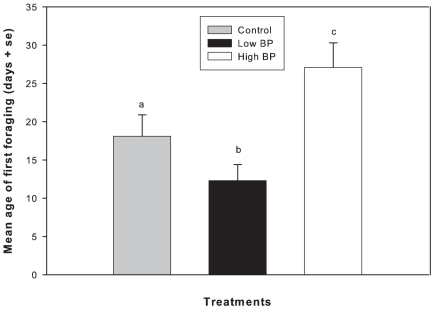
Mean age of first foraging in days (+SE). Different letters indicate significant difference between treatments. Cox regression was used to analyze the data, P<0.001.

There were significant differences in the age of first foraging among the three treatments (Cox regression χ^2^ = 29.3, P<0.001) (Fig. 7). High BP treatment colonies foraged at a significantly older age than controls and Low BP treated colonies. Overall, Low BP treatments foraged at a significantly younger age followed by controls and High BP treatments respectively.

## Discussion

This is the first study to directly connect division of labor associated with brood rearing to colony fitness (colony growth) through the mechanisms of brood pheromone modulation of brood rearing behavioral suites. Colonies receiving a relatively low amount of brood pheromone fielded a higher proportion of pollen foragers compared to controls and colonies treated with a relatively high amount of brood pheromone. Additionally, individual pollen foragers returned to the nest with larger pollen loads when exposed to a relatively low amount of brood pheromone compared to those treated with a relatively high amount of brood pheromone or solvent control. Individual bees initiated foraging at a younger age when treated with a low amount of brood pheromone relative to controls. Further, a higher proportion of bees within cohorts that were reared in colonies exposed to a low amount of BP were foragers, compared to those in control and high BP treated colonies. Combined, it can be inferred that Low BP treated colonies field a larger foraging population and those foragers are proportionally more likely to forage for pollen than non-pollen resources. Therefore, colonies respond to exposure to low amounts of BP by increasing the influx of pollen over time. As previously observed, this influx does not lead to increases in pollen stores [21], [22], [23], [24], [14]. Our study confirms that pollen stores are not significantly affected by brood pheromone treatment and the resultant behavioral modulation. Instead, colonies treated with a relatively low amount of BP reared significantly greater amounts of brood, as a direct result of increased pollen intake and consumption.

Interestingly hypopharyngeal gland protein content was significantly greater in Low BP and high BP treated bees compared to controls, in both cohorts 1 and 2 indicating an increased nutritional environment. In cohort 3 significant differences in the hypopharyngeal gland protein content were observed between all the three treatments, with High BP treatment having the highest protein content followed by Low BP and control treatments. High hypopharyngeal gland protein content is physiologically correlated with delayed ontogeny or older foraging ages. Our results support the speculations of Le Conte et al. (2001), that exposure to high BP dose delayed the behavioral development in bees, thus resulting in a lengthened nursing phase. The presence of greater number of non-foragers than foragers in the High BP treatment indicated that High BP dose extended the nursing phase in the bees such that these colonies fielded less number of foragers. Thus results of this study suggest that, by using different doses of brood pheromone to variably alter the division of brood rearing labor, we can affect colony growth and presumably fitness, over an extended period.

During the 30 day experimental period, brood pheromone modulated the division of labor and also significantly altered the growth trajectories of the experimental colonies. Following factors appear to have contributed for greater growth of the Low BP treatments 1) decrease in foraging age that resulted in fielding of more foragers to get resources 2) greater pollen foragers proportion compared to non-pollen foragers and 3) greater pollen foraging effort. Brood rearing in High BP and Control colonies was similar despite the fact that High BP treatments had a richer HP gland protein environment. Exploring the effects of different amounts of brood pheromone (linear dose-response) on nursing behaviors may provide a plausible answer to the above observation. One possible explanation is that a small amount of applied brood pheromone may induce foraging in anticipation of depletion of stores (as more pollen is needed for brood rearing), while a large amount of brood pheromone induces behaviors akin to a starvation scenario (more brood to be reared with less pollen), where emergency brood rearing behaviors, such as utilization of fat body stores and cannibalism of young larvae and eggs [25] occurs in order to improve the chances of survival, when the intake of food is inadequate compared to the task of rearing current brood populations. It is logical to expect that High BP dose should elicit higher pollen foraging effort i.e. higher pollen load weights and higher number of pollen foragers when compared to low BP dose, as High BP signals presence of greater number of larvae to rear. Our results do not appear to reflect the above expectation. We speculate that there exists an upper threshold for BP and when that threshold is reached a negative feedback mechanism kicks in and the colonies try to balance the ratio of adults to larvae, and greater number of bees stay inside the colony performing nursing duties thus decreasing the proportion of pollen foragers, and also may inhibit intensity of pollen foraging resulting in lower pollen load weights. Currently we don't know the threshold where this negative feedback kicks in and hence further studies are required to investigate this aspect using dose-response studies.

In conclusion, this study has demonstrated how division of labor associated with brood rearing affects honey bee rate of colony growth, a token of fitness, thus placing division of labor in an evolutionary context. This study has also shown that brood pheromone can be used as an empirical tool to uncover mechanisms of division of labor and address questions related to foraging strategies and colony fitness.

## Materials and Methods

This experiment was replicated 5 times using triple-cohort colonies [26], [11] during May 2006 at Texas A&M University apiary, College Station, TX (30° 6′ N; 96° 32′ W). A triple-cohort colony was comprised of three cohorts of 700 bees per cohort in their first, second and third week of adult life, respectively and a naturally mated queen. Beginning four weeks prior to establishing the triple cohort colony 2500 newly emerged bees were paint marked a unique color for each week and placed in a common foster colony for aging. A total of 2500 bees per target cohort ensured that at least 700 bees for the combined age and behavioral classes were easily found and collected. Cohort 1 comprised of 700 newly emerged adult bees less than 24 hours after emergence. Newly emerged bees were collected from combs of pupae placed in an incubator maintained at 32°C and 55% RH for 6 hours. Cohort 1 received a colored plastic number tag glued (BioQuip Products Inc. 1172, CA, USA) to the thorax and was the focal cohort for age of first foraging. Cohort 2 consisted of 700 nurse bees ranging in age from 8 to 11 days and selected from the brood nest area. Cohort 3 consisted of 700 foragers in their third week of adult life. Nurses and foragers were collected from their foster colony using a portable insect vacuum device [27].

On a weekly basis 50 newly emerged bees were added to the triple cohort colonies to simulate natural emergence of an established colony. Triple-cohort colonies have been recorded to demonstrate normal rates of behavioral development, with the benefit of a controlled adult demographic distribution [26], [11]. At the onset of the experiment each colony was provided with 1 frame of honey (1600 cm^2^), ¼ frame of pollen (400 cm^2^), and 2 frames of empty comb space (4800 cm^2^). Brood pheromone was applied to glass plates (500 cm^2^) and inserted between two brood frames.

There were three treatments as follows for 30 days: 1) BP dose of 336 µg per day that corresponds to 600 larval equivalents of synthetic brood pheromone 2) BP dose of 168 µg per day that corresponds to 300 larval equivalents of synthetic brood pheromone, and 3) solvent control. Treatments 1 and 2 represent high and low doses of brood pheromone, respectively. Empty comb space was added as necessary and equally to all treatments. The fatty acid ester blend of brood pheromone used here was as follows: 1% ethyl linoleate, 13% ethyl linolenate, 8% ethyl oleate, 3% ethyl palmitate, 7% ethyl stearate, 2% methyl linoleate, 21% methyl linolenate, 25% methyl oleate, 3% methyl palmitate, and 17% methyl stearate.

### Parameters measured

The ratio of pollen to non-pollen foragers was measured by daily counting the number of foragers of each type that entered colonies in a 5-minute period once in the morning and once in the afternoon. Daily observations of foraging activity began 24 hours after onset of the experiment. Every third day the comb area occupied by eggs, larvae, pupae, pollen, honey and empty space was measured with a metered grid [14].

Beginning on the third day, to the termination of the experiment, colony entrances were blocked with wire-mesh for 15 min intervals separated by at least 30 min to enable the capture of returning focal foragers. The entrances were blocked from 0800 h to 1700 h for a total of 4 h per day. Foragers were individually captured in small cylindrical wire cages. The identity of the captured foragers was recorded and the individuals released. Foragers were also classified as pollen or non-pollen foragers. At the termination of the experiment all number tagged bees were collected. Number of days from emergence to date of observation was used to estimate age of first foraging. Those that were not observed as foragers were categorized as censored cases in subsequent survival analysis.

Every week, 10 bees from each cohort were collected for hypopharyngeal gland protein analysis. The Bradford assay was used to estimate the hypopharyngeal gland protein content [28]. Bees that were sampled for hypopharyngeal gland protein analysis were also included as censored cases in the survival analysis data set.

### Statistical analyses

Contingency table analysis was used to analyze the ratio of pollen to non-pollen foragers observed and also to analyze proportion of foragers to non-foragers [29]. ANOVA was used to analyze pollen load weights and protein extractable from hypopharyngeal glands [29]. Brood and pollen areas were analyzed using repeated measures ANOVA. Normality was estimated by using normal probability plots. Data were log transformed prior to analysis to meet assumptions of ANOVA [29]. Homogeneity of variances was determined by Levene's test. Survival analysis was used to analyze age of first foraging data [30].

Bees were handled in compliance with current laws of the United States of America.
